# Effect of urban vs. remote settings on prehospital time and mortality in trauma patients in Norway: a national population-based study

**DOI:** 10.1186/s13049-023-01121-w

**Published:** 2023-10-05

**Authors:** Inger Marie Waal Nilsbakken, Mathias Cuevas-Østrem, Torben Wisborg, Stephen Sollid, Elisabeth Jeppesen

**Affiliations:** 1 Department of Research, Norwegian Air Ambulance Foundation, Oslo, Norway; 2 Faculty of Health Sciences, University of Stavanger, Stavanger, Norway; 3 Interprofessional rural research team – Finnmark, Faculty of Health Sciences, University of Tromsø – the Arctic University of Norway, Tromsø, Norway; 4 Norwegian National Advisory Unit on Trauma, Division of Emergencies and Critical Care, Oslo University Hospital, Oslo, Norway; 5 Hammerfest Hospital, Department of Anaesthesiology and Intensive Care, Finnmark Health Trust, Hammerfest, Norway; 6 Prehospital Division, Oslo University Hospital, Oslo, Norway; 7 Faculty of medicine, University of Oslo, Oslo, Norway; 8 Faculty of Health Studies, VID Specialized University, Oslo, Norway

**Keywords:** Trauma, Emergency medicine, Prehospital care, Trauma registries, Quality of healthcare, Epidemiology

## Abstract

**Background:**

Norway has a diverse population pattern and often long transport distances from injury sites to hospitals. Also, previous studies have found an increased risk of trauma-related mortality in remote areas in Norway. Studies on urban vs. remote differences on trauma outcomes from other countries are sparse and they report conflicting results.The aim of the present study was to investigate differences in prehospital time intervals in urban and remote areas in Norway and assess how prehospital time and urban vs. remote settings were associated with mortality in the Norwegian trauma population.

**Methods:**

We performed a population-based study of trauma cases included in the Norwegian Trauma Registry from 2015 to 2020. 28,988 patients met the inclusion criteria. Differences in study population characteristics and prehospital time intervals (response time, on-scene time and transport time) were analyzed. The Norwegian Centrality Index score was used for urban vs. remote classification. Descriptive statistics and relevant non-parametric tests with effect size measurements were used. A binary logistic regression model, adjusted for confounding factors, was performed.

**Results:**

The prehospital time intervals increased significantly from urban to remote areas.Adjusted for control variables we found a significant relationship between prolonged on-scene time and higher odds of mortality. Also, suburban areas compared with remote areas were associated with higher odds of mortality.

**Conclusion:**

In this nationwide study comparing prehospital time intervals in urban and remote areas, we found that prehospital time intervals in remote areas exceeded those in urban areas. Prolonged on-scene time was found to be associated with higher odds of mortality, but remoteness itself was not.

## Background

The topic of prehospital time has been widely studied and discussed for the last two decades, with a primary focus on the effect of different prehospital time intervals on mortality. The principle that shorter prehospital time intervals increase survival (i.e., the idea of the ‘golden hour’ [1]) has been advocated, but remains debated as studies report conflicting results regarding the relationship between prehospital time and mortality [2–7]. Several mediating and confounding factors, for example different trauma systems or trauma populations can influence the results. Also, various geographical challenges make a significant difference, such as urban-remote differences [3]. The literature on how urban-remote differences affect trauma mortality is, however, deficient.

A previous study from Norway on the influence of population density on trauma mortality [8], found that remote areas with low population density had higher mortality rates compared with urban areas. Also, the majority of deaths following trauma in remote areas occurred in the prehospital phase. This is in line with another study, also from Norway, on the effect of remoteness on mortality [9]. They found that patients in the most remote areas of Norway had an increased risk of dying following trauma compared with less remote and urban areas of Norway. These two studies, however, were conducted before the implementation of a trauma system in Norway and the establishment of a national trauma registry. In addition, the majority of deaths were caused by road traffic accidents, and a great deal has happened in the recent decade in terms of road safety. Finally, to our knowledge, no previous studies have looked into the effect of both prehospital time and urban vs. remote differences on trauma mortality in the Nordic countries. Thus, we aimed to investigate how urban vs. remote settings and prehospital time influenced trauma mortality by making use of data from the Norwegian trauma registry (NTR). More specifically, we used a comprehensive national trauma registry permitting a detailed investigation of prehospital time differences in urban and remote areas, and its influence on mortality.

The primary objective was to investigate differences in prehospital time intervals (response time, on-scene time and transport time) in urban, suburban and remote areas of Norway. The secondary objective was to assess the effect of urban-remote settings and prehospital time on trauma mortality.

We believe this study may be relevant for other countries with similar trauma systems and population patterns where remoteness is a challenge.

## Methods

### Design and settings

We performed a register-based study of trauma cases included in the NTR between 1 and 2015 and 31 December 2020. Norway has a population of 5.5 million people, with approximately 43% of the population living in urban areas, 43% in suburban areas and 14% living in remote areas [10, 11]. It is a high-income country with a publicly funded healthcare system. A national trauma plan has been developed and implemented, and includes all stages of the chain of survival, from accident site to rehabilitation [12, 13]. Furthermore, the Norwegian health and hospital plan has been developed to ensure a coherent system of emergency services in and outside hospitals throughout the country [14]. Thirty-four trauma units (TU) and four major trauma centers (MTC) receive and treat trauma patients, and report data to the NTR. All TU and MTC have 24/7 trauma team availability led by an advanced trauma life support-educated experienced resident or a surgical consultant. Calls made to the national medical emergency number (113) are evaluated by specially trained emergency medical communication center (EMCC) personnel using the ‘Norwegian Index for Medical Emergencies’ (Index), a criteria-based dispatch system for prehospital resources [15].

### Data sources and study cohort

The NTR is a national clinical quality registry containing information about injured patients in Norway from accident to rehabilitation (according to the Utstein template [16]). The NTR received formal status as a national medical quality register in 2006 [17]. All patients are registered with a waiver of consent. Injuries are coded according to the Abbreviated Injury Scale (AIS) manual (2005 version, updated in 2008 [18]) by certified nurse registrars. The NTR holds information about patients who meet the following inclusion criteria: admitted through trauma team activation (TTA), admitted without TTA but found to have penetrating injuries to head, neck, torso or extremities proximal to knee or elbow, head injury with AIS ≥ 3 or New Injury Severity Score (NISS) > 12, or patients who die at the scene of injury or during transport [17]. We excluded patients with injuries from drowning, inhalation, hypothermia and asphyxia without concomitant trauma, patients who presented to hospital via private vehicle, police vehicle or other/unknown, patients who were not registered with the EMCC, patients missing centrality index score and patients with prehospital time intervals we considered to be outliers (response time > 120 min, on-scene time < 5 min or > 120 min, or transport time < 5 min or > 360 min). Multiple registrations on the same patient (i.e., transfers) were counted only once. We chose to include patients with NISS = 0 for the descriptive statistics analysis as we wanted to investigate the trauma system and the prehospital phase where the patients were believed to be seriously injured. In the regression model we have excluded patients with NISS = 0.

### Data collection and management

**Fig. 1:**
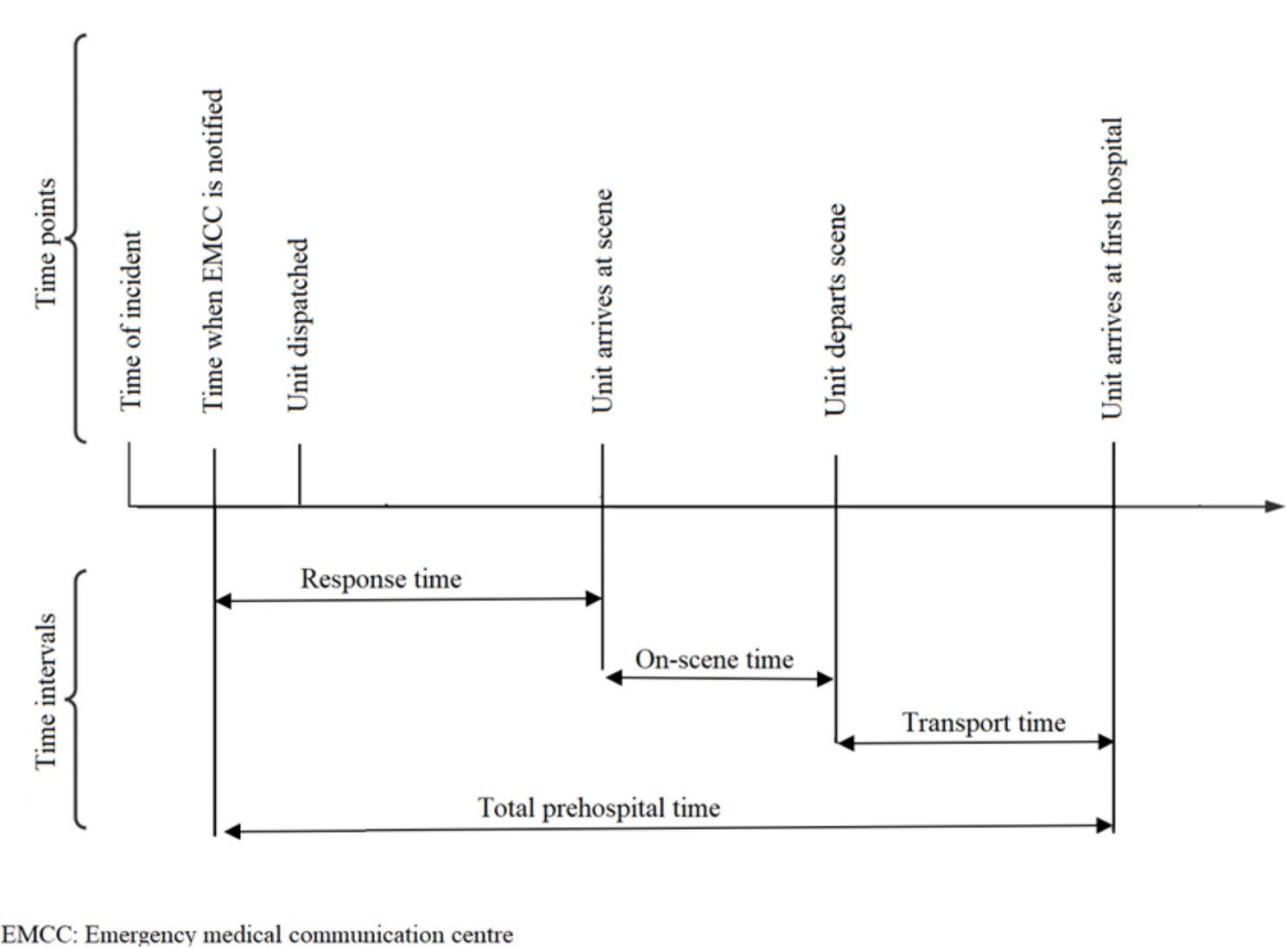
Time points and time intervals

Data collected from the NTR include time points from which we have calculated the prehospital time intervals for further analyses, illustrated in Fig. 1. Time cut-offs were applied to all prehospital time intervals (see exclusion criteria above).

Other variables collected from the NTR include transport type (ground ambulance, rotor-wing, fixed-wing), mortality (measured by 30-day mortality) and accident municipality for urban–remote classification. Rotor- and fixed-wing transport modes were merged into ‘air ambulance’, of which rotor wing constituted 97%. Patient characteristics included age, gender, injury mechanism, dominant injury, NISS, prehospital advanced airway management, whether or not the patient was trapped on the accident site, whether the patient was transported to a TU or MTC, and prehospital treatment level among the prehospital crew. The latter variable is only an indicator of the crews’ qualifications, and we do not know the amount of experience among prehospital crew. The injury mechanism variable was re-categorized from the original NTR definitions, where four traffic-related injuries (motor vehicle, motorcycle, pedestrian and other) were merged into ‘transport-related’, and shot by firearm, stabbed by sharp object, explosion injury and other were merged into ‘other’. Patients without a Norwegian ID number were registered as ‘missing age’. The variable coverage was high (see appendix).

### Measure of centrality: The centrality index of Norway

Statistics Norway’s centrality index (CI) provides a measure of the municipality’s centrality based on criteria such as travel time to workplaces and service functions [11]. Municipalities are categorized into six groups, where the proportion of inhabitants in each group is an important criterion for the classification [11]. Furthermore, the six CI groups are merged into urban (CI 1 and 2), suburban (CI 3 and 4) and remote (CI 5 and 6) areas. In 2020, a national municipality structure reform was accomplished, but we have used the original municipal division in this study.

### Statistical analysis

Registry data were analyzed using descriptive statistical methods including number, frequency (percentage) and median. Data were tested for normality with Kolmogorov–Smirnov tests. Differences in prehospital time intervals between the three centrality index groups were analyzed using Mann–Whitney U tests. Effect size was calculated to Mann–Whitney U with Cohen’s classification of effect sizes, where < 0.3 = small effect, between 0.3 and 0.5 = moderate effect and > 0.5 = large effect. A simple logistic regression model was performed to assess the effect of centrality index groups on mortality. Further, a forward stepwise logistic regression modeling strategy was applied to investigate the effects of prehospital time and centrality index groups on 30-day mortality, where we adjusted for control variables we believed would affect the results, including NISS, age, gender, injury mechanism and prehospital treatment level among the prehospital crew. All independent variables were tested for multicollinearity. Nagelkerke R^2^ was used to evaluate model improvement. A p-value of < 0.05 was considered to be statistically significant. All analyses were performed using SPSS v. 27.0 (IBM Company, Chicago, IL, USA).

## Results

**Fig. 2:**
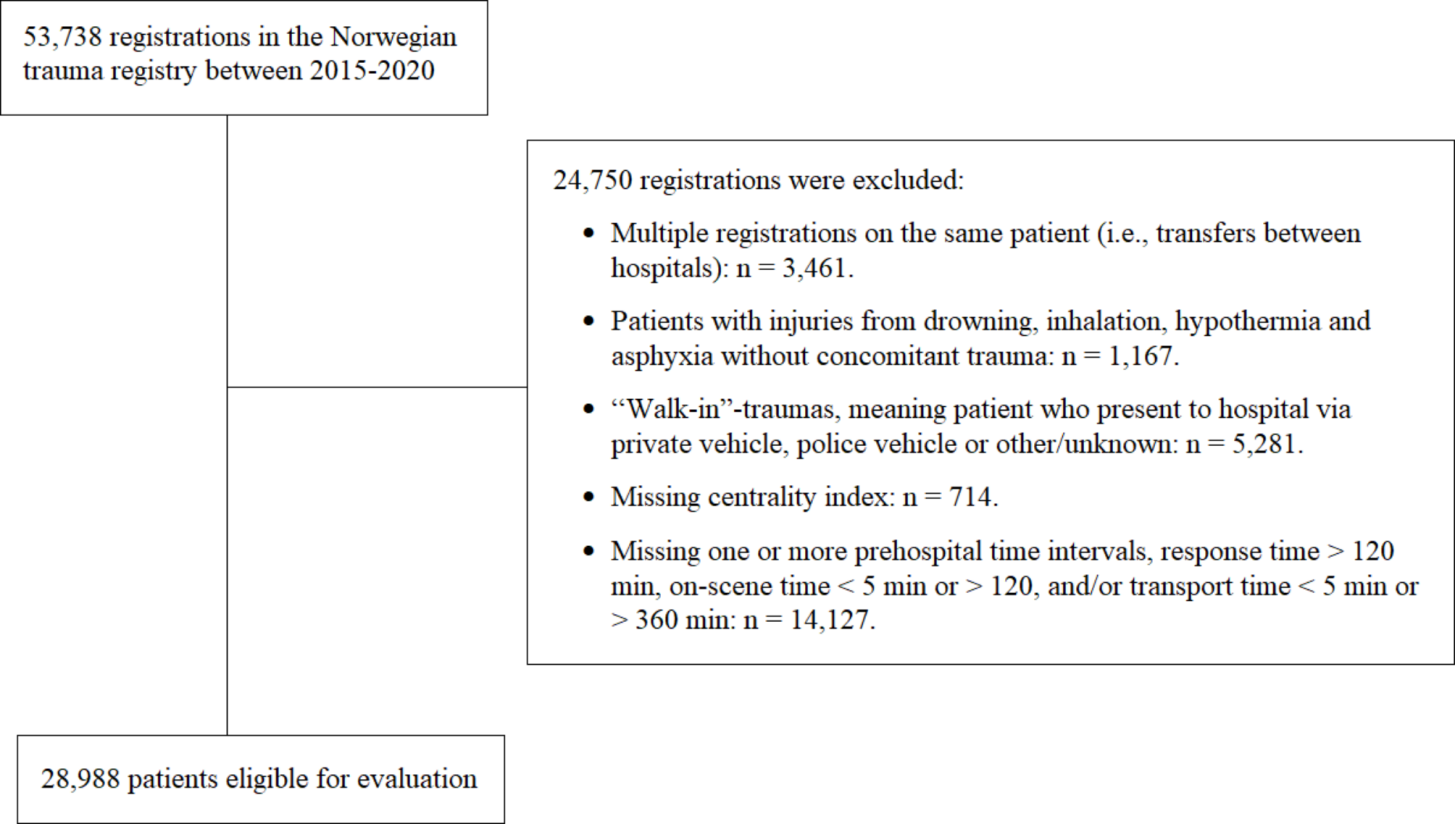
Flowchart of inclusion/exclusion

A total of 53,738 patients were registered in the NTR in the study period and 28,988 patients met the inclusion criteria (Fig. 2).

The included trauma patients had a median age of 42 (IQR 22, 62), 67% were male, transport-related injuries occurred most frequently (49%) and the median NISS was 5 (IQR 1, 13). 35% were injured in urban areas, 49% in suburban areas and 17% in remote areas.

### Study population characteristics

Study population characteristics are presented in Table 1. The proportion of male patients was approximately 2/3 in all areas. The age and NISS distribution across groups was similar. The proportion of patients who suffered low-energy fall was higher in urban areas compared with suburban and remote areas. The proportion of transport-related injuries was higher in suburban and remote areas compared with urban areas. The overall mortality rate was higher in urban areas compared with remote areas. The proportion of air ambulance transportations was higher in remote areas compared with both suburban and urban areas. The majority of patients in suburban and remote areas were transported to a TU. The proportion of patients receiving endotracheal intubation (ETI) was low in all areas (two per cent), but for patients with low Glasgow Coma Scores (GCS) (GCS < 9) in remote areas, the proportion of ETI was 47% compared with 27% in urban areas, and 26% in suburban areas. The proportion of patients trapped at the injury site was 4% in remote areas compared with 1% in urban areas, and 2% in suburban areas. The proportion of basic life-support (BLS) was approximately the same in all areas, while the proportion of advanced life-support (ALS) led by doctor was 34% in remote areas compared with 11% in urban areas, and 15% in suburban areas (Table 1).

**Table 1:** Patient characteristics

Variables			Urban	Suburban	Remote
Patients		Number	10,063	14,114	4,811
Age		Median (IQR)	44 (25, 63)	40 (21, 61)	41 (21, 61)
	0–15	Per cent	9%	10%	11%
	16–64	Per cent	69%	70%	71%
	≥ 65	Per cent	22%	19%	19%
Male		Per cent	66%	66%	70%
NISS		Median (IQR)	6 (1, 14)	5 (1, 12)	5 (2, 13)
	9–15	Per cent	21%	18%	21%
	> 15	Per cent	24%	19%	20%
GCS < 9		Per cent	8%	5%	3%
Dominant injury	Blunt	Per cent	93%	97%	98%
Injury mechanism distribution	Transport-related	Per cent	39%	54%	55%
	Low-energy fall	Per cent	20%	13%	11%
	High-energy fall	Per cent	24%	22%	23%
	Struck or hit by blunt object	Per cent	10%	7%	7%
	Other	Per cent	8%	5%	5%
Mortality rate*		Per cent	4%	3%	2%
Transport mode	Ground ambulance	Per cent	97%	89%	66%
	Air ambulance	Per cent	3%	11%	34%
First treatment hospital	TU	Per cent	40%	85%	77%
	MTC	Per cent	60%	15%	23%
First treatment hospital if NISS > 15	TU	Per cent	31%	73%	62%
	MTC	Per cent	69%	27%	38%
Proportion of patients with advanced airway management (AAM)	No AAM	Per cent	97%	98%	97%
	ETI	Per cent	2%	2%	2%
	Other/unknown	Per cent	0.4%	0.3%	0.7%
Proportion of patients with advanced airway management (AAM) if GCS < 9	No AAM	Per cent	69%	68%	42%
	ETI	Per cent	27%	26%	47%
	Other/unknown	Per cent	4%	6%	12%
Proportion of patients trapped at injury site		Per cent	1%	2%	4%
Prehospital treatment level	BLS	Per cent	14%	15%	13%
	ALS led by paramedic/ambulance crew	Per cent	75%	71%	53%
	ALS led by doctor	Per cent	11%	15%	34%

IQR = Interquartile range

NISS = New Injury Severity Score

GCS = Glasgow Outcome Score

*30-day mortality

TU = Trauma unit

MTC = Major trauma center

NISS = New injury severity score

ETI = Endotracheal tube intubation

GCS = Glasgow Outcome Scale

BLS = Basic life-support

ALS = Advanced life-support

**Fig. 3:**
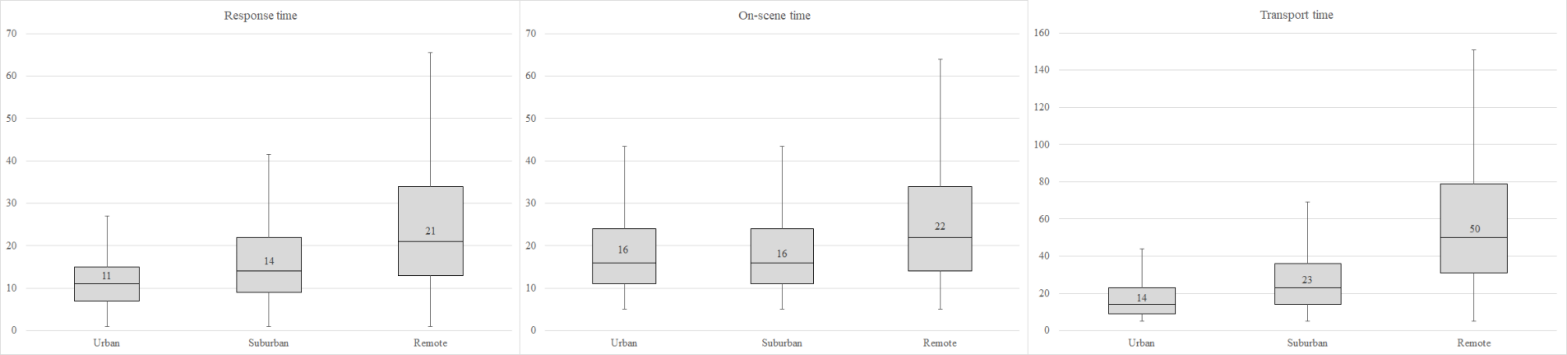
Panel of three figures showing response time, on-scene time and transport time (min). Boxplots show median time with interquartile ranges in in urban, suburban and remote areas

We found that prehospital time intervals (response time, on-scene time and transport time) in remote areas exceeded those in urban areas (Fig. 3), and there was a significant difference in prehospital time intervals between all areas except for on-scene time between urban and suburban areas (see appendix for information regarding test statistics). The effect size measurements for response time were medium for the difference between urban and remote areas, and small for the differences between the remaining areas. The effect size measurements for on-scene time were small for the difference between all areas. For transport time, the effect size measurements were medium for the difference between urban and suburban areas and suburban and remote areas, and large for the difference between urban and remote areas (see appendix for information regarding test statistics).

### Mortality analysis

We assessed the relationship between urban vs. remote differences and mortality. Compared with remote areas both urban and suburban areas were associated with higher odds of mortality, but centrality index explained little of the variation in mortality, described by a Nagelkerke R^2^ of 0.08 (Table 2).

**Table 2:** Simple logistic regression table where patients with NISS = 0 were excluded

	Model 1		
Independent variables	Odds ratio	95% CI	p-value
Centrality index			
Urban	2.30	1.79–2.96	< 0.001
Suburban	1.68	1.31–2.17	< 0.001
Remote	Ref.	Ref.	Ref.

CI = Confidence interval.

NISS = New Injury Severity Score.

Nagelkerke R^2^ for the model was 0.08.

The model improved when prehospital time intervals and our control variables gender, age, NISS, injury mechanism and prehospital treatment level among the prehospital crew were added to the model. Nagelkerke R^2^ for the final model was 0.46. In the final model, only suburban areas compared with remote areas remained significantly associated with higher odds of mortality. We found that prolonged on-scene time was associated with higher odds of mortality and prolonged transport time was associated with lower odds of mortality. An increase in age and an increase in NISS were both associated with higher odds of mortality. A prehospital treatment level among the prehospital crew of “ALS led by doctor” was associated with higher odds of mortality (Table 3).

**Table 3:** Adjusted logistic regression table where patients with NISS = 0 were excluded

	Model 2		
Independent variables	Odds ratio	95% CI	p-value
Centrality index			
Urban	1.28	0.94–1.76	0.120
Suburban	1.36	1.00-1.84	0.049
Remote	Ref.	Ref.	Ref.
Response time	1.00	0.99-1.00	0.356
On-scene time	1.01	1.00-1.01	0.049
Transport time	0.99	0.99-1.00	< 0.001
Age	1.07	1.06–1.08	< 0.001
NISS	1.10	1.09–1.10	< 0.001
Gender			
Male	1.15	0.96–1.38	0.130
Female	Ref.	Ref.	Ref.
Prehospital treatment level among the prehospital crew			
BLS	Ref.	Ref.	Ref.
ALS	1.13	0.86–1.47	0.380
ALS led by doctor	2.07	1.50–2.87	0.001

CI = Confidence interval

NISS = New Injury Severity Score

BLS = Basic life-support

ALS = Advanced life-support

The model is adjusted for injury mechanism.

Nagelkerke R^2^ for the model was 0.46.

## Discussion

The amount of studies conducted on the relationship between prehospital time and mortality is comprehensive, although not conclusive. Mortality analyzes with urban vs. remote differences are also deficient, something we have attempted to address with this study. In our unadjusted model we found that both urban and suburban areas were independently associated with mortality compared with remote areas. However, centrality index explained little of the variation in mortality. In our final model, about half of the variation in mortality could be explained by our independent variables (centrality index, prehospital time intervals, gender, NISS, age and prehospital treatment level among the prehospital crew), meaning there were other unknown factors that explained more of the outcome than our model. This is not surprising, given that the prehospital treatment is only a part of the treatment-chain that the patient meets from injury to rehabilitation, and that many other factors, including the intrahospital treatment, affect the overall 30-day survival rate.

In our adjusted model, on-scene time and transport time were found to be significantly associated with mortality: While prolonged on-scene time was associated with a higher odds of mortality, prolonged transport time was associated with higher odds of survival. Our findings regarding the relationship between prolonged on-scene time and increased odds of mortality is in line with a previous study [6]. However, they only investigated patients with moderate and severe trauma. In contrast, a recent systematic review [3] on the relationship between prehospital time and mortality, the majority of the included studies found no association between time spent at the accident site and mortality, and they concluded that it seemed longer on-scene time increased odds of survival. Regarding transport time, two of the studies included in the systematic review [19, 20], found an association between mortality and shorter transport time. This is similar to our results, where we found an association between prolonged transport time and reduced odds of mortality. .

There has been a tendency towards centralizing hospital functions which has resulted in fewer hospitals with trauma functions over the last two decades. In 2002, 52 hospitals in Norway received and treated trauma patients; today, there are 38. This has resulted in longer travel distances for emergency medical services, which is reflected in the long transport time intervals in remote areas. The long distances are not due to the vast majority of patients being transported directly to TC, as the majority of patients in suburban and remote areas are transported to TUs, also patients with severe injury (NISS > 15) (Table 1). The organization of ambulance stations involves a difficult compromise between shortest possible response time and quality of service. Long distances increases response time intervals, but additional ambulance stations in remote areas with few assignments may in turn lead to challenges in maintaining competence and difficulties in recruiting [14].

Longer response time and transport time intervals in remote areas compared to urban areas can be explained by the mentioned trauma system configuration where both hospitals and emergency medical services are localized in more densely populated areas. What cannot be explained by this, however, was the prolonged on-scene time intervals in remote areas compared to urban areas. Previous studies on the association between prolonged on-scene time and prehospital stabilizing procedures report conflicting results [21] [22].In our study we found that the proportion of patients who received endotracheal intubation (ETI) was similar in all areas (two per cent). However, patients with low GCS scores (< 9) in remote areas had ETI performed nearly twice as often as patients in urban and suburban areas (Table 1). One study from 2018 [23] reported that one of the parameters that correlated with prehospital intubation was distance from scene to hospital. Current Scandinavian guidelines also state that with low GCS scores and a compromised airway, an artificial airway should be established for long prehospital transports [24]. Thus, the large proportion of endotracheal intubation among patients with low GCS scores in remote areas can be explained by longer transport distances and this might also be a partial explanation for prolonged on-scene time in remote areas. However, relying solely on GCS scores as a single factor for considering ETI in trauma patients is not optimal. Various indications necessitate ETI beyond just low GCS scores, including facial, neck, and cheat trauma, where securing the airway often becomes necessary. Furthermore, we found that the proportion of patients who were trapped at the accident site was four times higher in remote areas compared with urban areas (4% vs. 1%). Prolonged on-scene time in remote areas might partially be explained by difficulties gaining access to the patient. Because of the entrapment, these patients may also be more severely injured. More severe injury in patients with prolonged on-scene time might explain the association between on-scene time and mortality.

The proportion of patients who were met by a doctor at the accident site in remote areas was more than twice compared to patients in urban and suburban areas. However, the variable prehospital treatment level among the prehospital crew is only an indicator of the crews qualifications, and we do not know the amount of experience among these. In our regression analysis, we found that patients who received on-scene medical attention from a doctor had increased mortality odds. Nevertheless, it’s important to consider that when the EMCC operator receives the emergency call, the decision to dispatch a doctor to the accident site is contingent upon their assessment of situation’s severity, signalling the requirement for a doctors expertise. Consequently, we believe that receiving prehospital care from a doctor is not inherently linked to a higher mortality risk. Instead, the connection lies in the severity of injuries necessitating a doctors presence at the scene, which in turn is associated with an increased risk of mortality. Also, a larger proportion of patients were transported by air ambulance from remote areas compared with suburban and urban areas. One explanation may be that helicopters have difficulty accessing densely populated areas; another may be that ground ambulances stationed nearby reach the patient more rapidly. On the other hand, a widespread use of air ambulances among suspected severely injured patients in remote areas might explain the high prehospital treatment level found among these patients.

### Limitations

Due to the observational design of the study, some limitations must be addressed, as it is prone to bias and confounding, and cannot be used to demonstrate causality. In large registry studies, type 1 errors can occur. Attempts have been made to take this into account by not only reporting statistical significance with p-values, but also by calculating and reporting effect size. Registry data can also lead to selection bias. Our results are based on the data in the NTR.

## Conclusion

Prehospital time intervals in remote areas exceeded those in urban and suburban areas. Furthermore, we found an increased odds of mortality in patients with prolonged on-scene time. Although prolonged on-scene time was more common in remote areas, remoteness itself was not associated with mortality.

## Appendix

**Table:** Variable coverage

Variables	Valid, n	Valid, per cent
Age	28,697	99%
Gender	28,988	100%
NISS	28,887	100%
Dominant injury	28,814	99%
Injury mechanism	28,809	99%
Mortality	28,487	98%
Prehospital airway management	28,913	100%
Prehospital treatment level	28,746	99%
GCS	26,910	93%

**Table:** Test statistics

		Mann-Whitney U	Sig.	Z	N	r	r effect size
Response time	Urban-Suburban	50863288.00	< 0.001	-37.723	24,177	0.2	Small
	Urban-Remote	11410026.00	< 0.001	-52.310	14,874	0.4	Medium
	Suburban-Remote	23641379.50	< 0.001	-31.528	18,925	0.2	Small
On-scene time	Urban-Suburban	70950697.000	0.9	-0.120	24,177	0.0	Small
	Urban-Remote	17746217.50	< 0.001	-26.389	14,874	0.2	Small
	Suburban-Remote	25109018.500	< 0.001	-27.039	18,925	0.2	Small
Transport time	Urban-Suburban	49125245.000	< 0.001	-40.940	24,177	0.3	Moderate
	Urban-Remote	7860490.500	< 0.001	-66.756	14,874	0.5	Large
	Suburban-Remote	14824378.500	< 0.001	-58.459	18,925	0.4	Moderate

## Data Availability

The data can be accessed through The Norwegian Trauma Registry by application.

## Abbreviations

NTR: Norwegian trauma registry
TU: Trauma units
MTC: Major trauma centres
EMCC: Emergency medical communications center
AIS: Abbreviated injury scale
TTA: Trauma team activation
NISS: New injury severity score
CI: Centrality index
ETI: Endotracheal intubation
GCS: Glasgow Outcome Score
BLS: Basic life-support
ALS: Advanced life-support
